# Renal Function Trajectories in Patients with Prior Improved eGFR Slopes and Risk of Death

**DOI:** 10.1371/journal.pone.0149283

**Published:** 2016-02-22

**Authors:** Yan Xie, Benjamin Bowe, Hong Xian, Sumitra Balasubramanian, Ziyad Al-Aly

**Affiliations:** 1 Clinical Epidemiology Center, VA Saint Louis Health Care System, Saint Louis, Missouri, United States of America; 2 Department of Biostatistics, College for Public Health and Social Justice, Saint Louis University, Saint Louis, Missouri, United States of America; 3 Division of Nephrology, Department of Medicine, VA Saint Louis Health Care System, Saint Louis, Missouri, United States of America; Ichan School of Medicine at Mount Sinai, UNITED STATES

## Abstract

**Background:**

Multiple prior studies demonstrated that patients with early Chronic Kidney Disease (CKD) and positive estimated Glomerular Filtration Rate (eGFR) slopes experience increased risk of death. We sought to characterize patients with positive eGFR slopes, examine the renal function trajectory that follows the time period where positive slope is observed, and examine the association between different trajectories and risk of death.

**Methods and Findings:**

We built a cohort of 204,132 United States veterans with early CKD stage 3; eGFR slopes were defined based on Bayesian mixed-effects models using outpatient eGFR measurements between October 1999 and September 2004; to build renal function trajectories, patients were followed longitudinally thereafter (from October 2004) until September 2013. There were 41,410 (20.29%) patients with positive eGFR slope and they exhibited increased risk of death compared to patients with stable eGFR slope (HR = 1.33, CI:1.31–1.35). There was an inverse graded association between severity of albuminuria and the odds of positive eGFR slope (OR = 0.94, CI:0.90–0.98, and OR = 0.76, CI:0.69–0.84 for microalbuminuria and albuminuria; respectively). Following the time period where positive eGFR slope is observed, we characterized 4 trajectory phenotypes: high eGFR intercept and positive trajectory (HIPT) (12.42%), intermediate intercept and mild negative trajectory (IIMNT) (60.04%), low intercept and fast negative trajectory (LIFNT)(23.33%), and high intercept and fast negative trajectory (HIFNT) (4.20%). Compared to IIMNT (reference group), HIPT is associated with younger age, dementia, HIV, chronic lung disease, peripheral artery disease, weight loss, and inversely associated with albuminuria; LIFNT and HIFNT were associated with diabetes, hypertension, cardiovascular disease, peripheral artery disease, and albuminuria. The risk of death at 9 years was lowest in IIMNT (HR = 1.12, CI:1.09–1.14), highest in HIPT (HR = 1.71, CI:1.63–1.79), and intermediate in LIFNT (HR = 1.36, CI:1.32–1.40) and HIFNT (HR = 1.56, CI:1.45–1.68).

**Conclusions:**

Our results demonstrate that patients with positive eGFR slopes, when followed over longer period of time, follow 4 distinct trajectory phenotypes that have distinct demographic and clinical correlates and are differentially associated with risk of death.

## Introduction

Early Chronic Kidney Disease (CKD) progresses in some but not all patients; and some do experience improvement in kidney function over time [1]. While decline in kidney function heralds a host of adverse outcomes, improvement in kidney function in general is expected to be associated with favorable outcomes [2]. Multiple prior observations suggest that patients who experience improvement in estimated Glomerular Filtration Rate (eGFR) over time, or positive eGFR slopes, exhibit an increased risk of adverse events or death. Perkins et al. observed that compared to patients with stable kidney function, those with increasing eGFR over time had an increased risk of death [3]. Matsushita et al. also reported that increasing eGFR is associated with increased risk of adverse outcomes [4]. Others reported that, compared to patients with age-commensurate decline in kidney function, patients with non-declining kidney function (rate of eGFR change >0 ml/min per 1.73 m^2^ per year) exhibited a trend toward increased risk of death [5, 6].

It has been hypothesized that increased mortality in patients with improved eGFR may be the result of a decrease in muscle mass secondary to severe illnesses, thus are at higher risk of death. Studies that took into consideration parameters related to weight and surrogate markers for nutritional status did not yield a full explanation. Turin and Hemmelgarn, on more than one occasion, eloquently discussed the need for studies to better characterize predictors of positive eGFR slopes, and to further elucidate the prognostic implications of improved kidney function [2, 7].

While there is preponderant literature on CKD progression, much less is known about those patients with positive eGFR slope, and while the association between positive eGFR slope and risk of death is now well documented, it remains counterintuitive, and poorly understood [7]. Our understanding of the renal function trajectory that follows the period where improved kidney function is observed is also very limited; and whether patients-with prior positive eGFR slope-experience further improvement, a plateau, or some degree of decline is not known. Furthermore, the relationship between different trajectory phenotypes and risk of death has not been examined.

In this manuscript, we sought to characterize those patients who experience improved chronic eGFR slopes, determine the renal function trajectory of these patients following the period of time where eGFR improvement is observed, and examine the association between type of renal function trajectory and risk of death.

## Methods

### Patients

**Fig 1:**
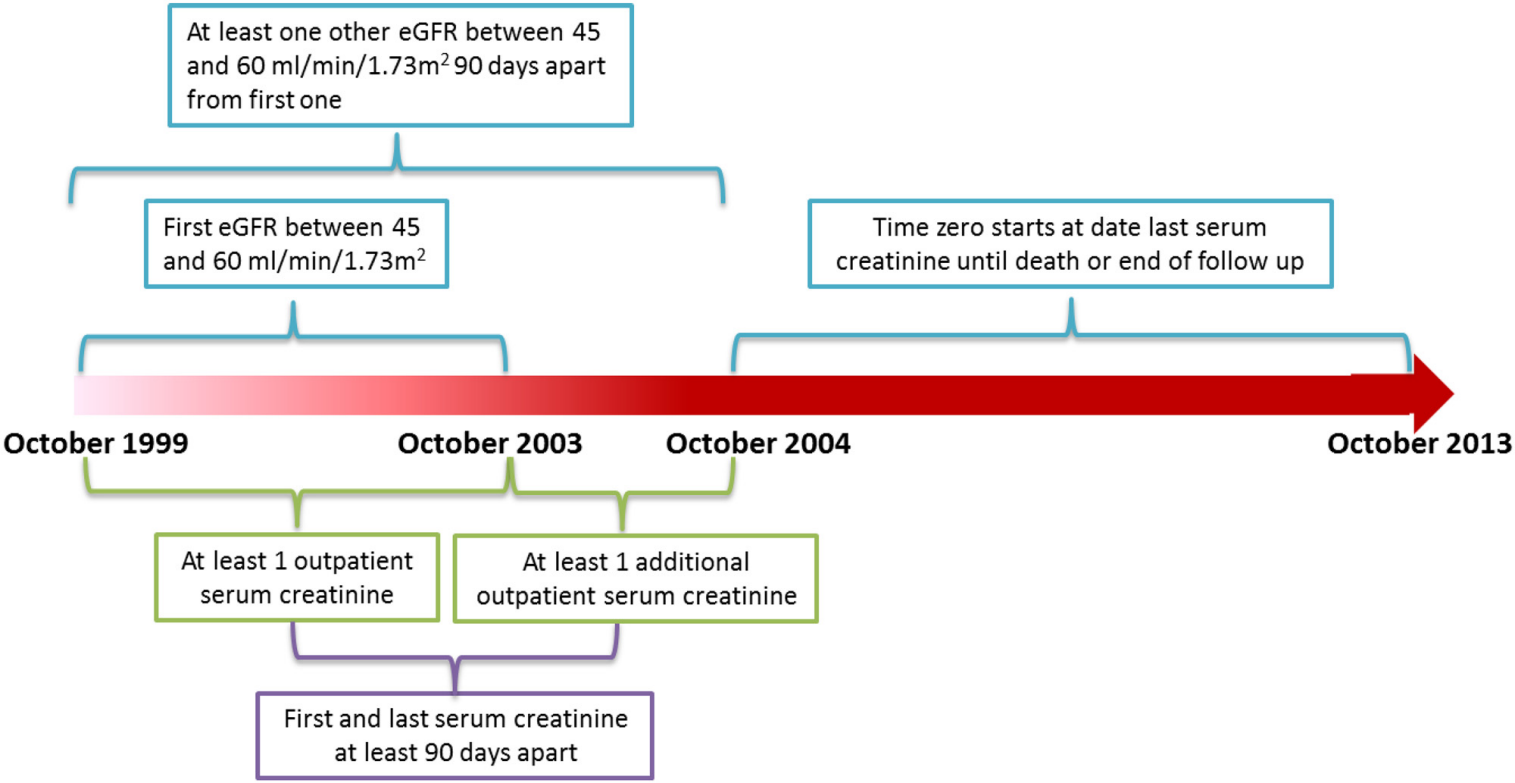
Cohort design and timeline.

We used administrative data from the United States Department of Veterans Affairs (VA) to build a retrospective longitudinal cohort of patients with early CKD stage 3. We identified United States veterans who have at least one estimated glomerular filtration rate (eGFR) between October 1, 1999 and September 30, 2003 and an additional eGFR between October 1, 2003 and September 30, 2004 (n = 2,606,981). Subjects were included in the cohort if the initial eGFR was between 60 and 45 ml/min/1.73 m^2^ and they had another eGFR between 60 and 45 ml/min/1.73 m^2^ separated by at least 90 days from the initial eGFR (n = 205,430). Subjects were excluded if they had received kidney transplantation, had undergone at least one session of dialysis before time zero (the time of the last eGFR measurement) yielding an analytic cohort of 204,132. Subjects were followed longitudinally until death or end of study follow up on September 2013 (Fig 1). The study (#1163689) was approved by the Institutional Review Board (IRB) of the VA Saint Louis Health Care System, Saint Louis, MO. A waiver of informed consent was approved by the IRB. Data was anonymized and de-identified prior to analysis.

### Data Sources

We used Department of Veterans Affairs databases including inpatient and outpatient medical SAS datasets (that include utilization data related to all inpatient and outpatient encounters within the VA system) to ascertain detailed patient demographic characteristics and comorbidity information based on Current Procedural Terminology (CPT) codes, and ICD-9-CM diagnostic and procedure codes associated with inpatient and outpatient encounters [8–11]. The VA Managerial Cost Accounting System Laboratory Results file (a comprehensive database that includes VA-wide results for selected laboratory tests obtained in the clinical setting) provided information on outpatient serum creatinine measurements and other laboratory variables [8, 9, 12]. The VA Vital Status and Beneficiary Identification Records Locator Subsystem (BIRLS) files provided demographic characteristics and death follow-up through September 30, 2013 [8, 9].

### Statistical Analysis

For the primary analysis, outpatient eGFR was calculated using the abbreviated 4-variable Chronic Kidney Disease Epidemiology Collaboration (CKD-EPI) equation based on age, gender, race, and serum creatinine concentration [13]. In the primary analyses, the time period for the assessment of eGFR change started on October 1, 1999 to September 30, 2004; the time period for assessment of eGFR trajectories started on October 1, 2004 until September 30, 2013.

#### Analysis of eGFR slope

While Ordinary Least Square (OLS) analysis is considered a valid method for estimating eGFR slope, due to eGFR variability that may not be truly reflective of change in kidney function, OLS tends to overestimate the proportion of patients with true long-term positive slopes [1, 5]. In order to control for this bias (eGFR changes due to variability in each measure); Bayesian linear mixed effect model was used to define each patient’s eGFR change. The likelihood function of patient’s eGFR followed a normal distribution. The hierarchical prior with three levels was used. Mean of the normal distribution equal to a time related linear regression and variance proportional to the mean eGFR level. Intercept and coefficient of the time related linear regression followed a bivariate normal distribution. Parameters of the bivariate normal distribution consisted of two normal distributions as its mean and a Wishart distribution as its variance [1]. Parameters for prior distributions were estimated from previous analyses [14]. Based on the Bayesian model, each patient’s mean posterior coefficient for independent time variable was considered as their eGFR change over time. Patients who had more than 95% chance to have a coefficient larger than 0 based on 5000 times of Markov Chain Monte Carlo iterations were considered as having eGFR improvement, patients with more than 95% chance of having a coefficient less than -1 were considered as having eGFR decline, while all other patients were considered to have normal eGFR progression. Distribution of eGFR slopes is presented in S1 Fig.

#### Trajectory analysis

For patients with improved kidney function before cohort entry (positive eGFR slope before T0), we undertook a group based trajectory modeling analysis using eGFR from October 2004 to September 2013 [15, 16]. In order to account for non-ignorable missing values, a Pattern-Mixture Latent Trajectory Model including a time independent covariate indicating whether patient had an eGFR measurement at the year of death was used [16–18]. In order to account for the variability of eGFR measurement frequency and timing, each patient’s annual average eGFR was computed and used in the trajectory model [19], and a time-varying covariate indicating number of eGFR measurements within each year was included in the model. Accepting N as Number of trajectories was decided based on 2 criteria: a) if Bayesian Information Criterion in N trajectory groupings is less then N-1 trajectory groupings; and b) if in N trajectories the average posterior probabilities of trajectory membership for all people assigned to that trajectory exceed 70% [15–17, 20].

Cohort entry time (T0) for the survival analyses started on last eGFR measure on or before September 30, 2004, until censorship or end of follow up on September 30, 2013. We built multivariate Cox regression models to examine the hazard of death for people with different eGFR progression before T0, and for people with increasing eGFR before T0 having different trajectories after T0. We used multinomial logistic regression to detect factors that affect eGFR progression before T0 and trajectories after T0. In survival analyses, a 95% confidence interval (CI) of a hazard ratio (HR) that does not include unity was considered statistically significant. In all analyses a p-value of 0.05 or less was considered statistically significant. Analyses were performed using SAS Enterprise Guide version 6.1, and SAS 9.2 (SAS Institute, Cary, NC).

### Outcome

The primary outcome was time from T0 to death. Death data were ascertained through September 30, 2013.

### Covariates

Baseline period was defined as the 5 year preceding cohort entry (time 0). Baseline covariates were captured during the baseline period. Covariates included initial eGFR, number of eGFR measurements, age, race, gender, and diabetes mellitus, hypertension, cardiovascular disease, hyperlipidemia, peripheral artery disease, cerebrovascular disease, chronic lung disease, hepatitis C, HIV, and dementia. Race/ethnicity was categorized as white, black, and other (Latino, Asian, Native American, or other racial/ethnic minority groups). Comorbidities were assigned on the basis of relevant ICD-9-CM diagnostic and procedures codes and CPT codes in the VA Medical SAS datasets [21]. Annual percentage change in eGFR calculated using the formula: [(T0 eGFR-initial eGFR)/ initial eGFR)]/[(T0 date-initial date)/365.25]*100. Data on weight was available for n = 185,508. Average annual percentage change in weight was defined as difference of subject’s first and last weight before T0 divided by time between these two measurements and also first weight value. Data on microalbuminuria was available on n = 63,021, micro-albumin/creatinine ratio <20, 20–300, and >300 mg/g was categorized as normal, microalbuminuria, and albuminuria; respectively.

### Sensitivity analyses

We evaluated the consistency of study findings by undertaking a number of sensitivity analyses where we rebuilt the cohort to a) widen the period of time where eGFR slope is captured to 9 years (October 1, 1999 to September 30, 2008), b) liberalized inclusion criteria to require only the initial eGFR to be between 60 and 45 ml/min/1.73 m^2^, c) we excluded all patients with AKI or hospitalization during a wash out period of 2 years before positive eGFR slope be assessed. d) we undertook analyses where we excluded or censored patients with kidney transplantation, dialysis and ESRD where ESRD was defined as outpatient eGFR less than 15 ml/min/1.73m^2^ and e) we also performed analyses where we included patients with 5 or more eGFR measurements before T0.

## Results

The demographic and clinical characteristics of the overall cohort and of those with improved, stable, and declining eGFR slope are presented in Table 1. There were 204,132 patients in the overall cohort, and 41,410 (20.29%), 111,554 (54.65%), and 51,168 (25.07%) patients in the groups with improved, stable, and declining eGFR slope; respectively. The majority of patients were of white race, and male. The prevalence of comorbid conditions such as hypertension, diabetes mellitus, cardiovascular disease, and chronic lung disease was high.

**Table 1:** Demographic and clinical characteristics of those with improved, stable, and declining eGFR slope.

	Overall	Improved eGFR slope	Stable eGFR slope	Declining eGFR slope
Number (%)	204,132	41,410 (20.29)	111,554 (54.65)	51,168 (25.07)
Age (SD)	70.93 (7.82)	69.48 (8.53)	71.12 (7.71)	71.68 (7.28)
Race				
White (%)	178,774 (87.58)	35,481 (85.68)	98,926 (88.68)	44,367 (86.71)
Black (%)	21,785 (10.67)	5,205 (12.57)	10,741 (9.63)	5,839 (11.41)
Other (%)	3,573 (1.75)	724 (1.75)	1,887 (1.69)	962 (1.88)
Male Gender (%)	195,597 (95.82)	39,293 (94.89)	106,822 (95.76)	49,482 (96.70)
Cerebrovascular Disease (%)	2,070 (1.01)	400 (0.97)	873 (0.78)	797 (1.56)
Cardiovascular Disease (%)	99,842 (48.91)	19,772 (47.75)	49,922 (44.75)	30,148 (58.92)
Dementia (%)	9,447 (4.63)	2,212 (5.34)	4,346 (3.90)	2,889 (5.65)
Diabetes Mellitus (%)	74,972 (36.73)	14,639 (35.35)	34,708 (31.11)	25,625 (50.08)
Hepatitis C (%)	3,025 (1.48)	816 (1.97)	1,216 (1.09)	993 (1.94)
HIV (%)	12,474 (6.11)	2,991 (7.22)	5,640 (5.06)	3,843 (7.51)
Hypertension (%)	174,481 (85.47)	35,073 (84.70)	92,335 (82.77)	47,073 (92.00)
Hyperlipidemia (%)	148,537 (72.77)	29,554 (71.37)	80,107 (71.81)	38,876 (75.98)
Chronic Lung Disease (%)	49,848 (24.42)	11,283 (27.25)	23,921 (21.44)	14,644 (28.62)
Peripheral Artery Disease (%)	11,045 (5.41)	2,166 (5.23)	4,486 (4.02)	4,393 (8.59)
Death (%)	89,156 (43.68)	17,519 (42.31)	42,350 (37.96)	29,287 (57.24)
Average eGFR slope before T0 ml/min/1.73m2/year * (SD)	-0.57 (3.84)	4.01 (3.36)	-0.36 (1.67)	-4.76 (2.99)
Average initial eGFR in ml/min/1.73m2 (SD)	53.90 (4.17)	53.18 (4.30)	54.05 (4.10)	54.17 (4.14)
Average T0 eGFR in ml/min/1.73m2 (SD)	52.71 (11.22)	64.18 (10.29)	53.50 (6.72)	41.70 (9.44)
Median duration between initial and T0 eGFR in years (IQR)	3.38 (2.18, 4.23)	3.51 (2.43, 4.26)	3.11 (1.94, 4.08)	3.78 (2.70, 4.42)
Median number of eGFR measure before T0 (IQR)	7 (4, 12)	8 (5, 12)	6 (4, 10)	10 (6, 16)
Median number of eGFR measure after T0 (IQR)	13 (7, 21)	13 (7, 21)	12 (6, 20)	13 (6, 23)
Weight				
Number	185,508	38,997	99,376	47,135
Average Annual change lb/year (SD)	-0.59 (10.87)	-1.20 (7.01)	-0.45 (9.42)	-0.39 (15.39)
Median Annual percentage change (IQR)	-0.26% (-1.44%, -0.90%)	-0.47% (-1.76%, -0.72%)	-0.22% (-1.34%, -0.89%)	-0.18% (-1.40%, -1.07%)
Micro albumin / Creatinine Ratio				
Number	63,021	12,792	33,094	17,135
Normal (%)	35,369 (56.12)	7,716 (60.32)	19,531 (59.02)	8,122 (47.40)
Mild (%)	23,376 (37.09)	4,455 (34.83)	11,781 (35.60)	7,140 (41.67)
Severe (%)	4,276 (6.79)	621 (4.85)	1,782 (5.38)	1,873 (10.93)

*Reflects the rate of change of eGFR per year calculated using a Bayesian mixed effect model. The slope of the regression line describes the rate of change in kidney function (eGFR) over time.

There were 17,519 (42.31%), 42,350 (37.96%), 29,287 (57.24%) deaths among patients with improved, stable, and declining eGFR slope; respectively. Compared to patients with stable eGFR, those with improved eGFR had increased risk of death at 1, 3, 5, and 9 years (Table 2). In models that additionally adjust for weight, and albuminuria, the risk was mildly attenuated, but remained significant (Table 2). The results were consistent in models that adjust for initial eGFR-instead of eGFR at T0 (S1 Table). In models that adjust for eGFR changes after time zero, prior positive eGFR slope remained significantly associated with increased risk of death (HR = 1.30; CI: 1.27–1.32). There was no significant interaction between presence or severity of albuminuria and positive eGFR slope. Presence and severity of albuminuria attentuated the association between declining eGFR slope and the risk of death.

**Table 2:** Change in kidney function and the risk of death. Model 1 adjusted for age, race, gender, diabetes mellitus, hypertension, cardiovascular disease, hyperlipidemia, peripheral artery disease, cerebrovascular disease, chronic lung disease, hepatitis C, HIV, dementia and eGFR at time of cohort entry (T0). Reference group is patients with stable kidney function (eGFR slope).

	1-year HR (CI)	3-year HR (CI)	5-year HR (CI)	9-year HR (CI)
Model 1				
Improved eGFR slope	2.04 (1.94–2.15)	1.59 (1.55–1.64)	1.43 (1.40–1.46)	1.33 (1.31–1.35)
Declining eGFR slope	2.16 (2.05–2.26)	1.58 (1.53–1.63)	1.46 (1.42–1.50)	1.35 (1.32–1.37)
Model 1 + annual percentage weight change				
Improved eGFR slope	1.76 (1.63–1.89)	1.45 (1.39–1.50)	1.38 (1.34–1.42)	1.30 (1.27–1.32)
Declining eGFR slope	1.57 (1.47–1.68)	1.39 (1.34–1.43)	1.30 (1.27–1.34)	1.26 (1.24–1.28)
Model 1 + annual percentage weight change + albuminuria				
Improved eGFR slope	1.72 (1.38–2.14)	1.39 (1.26–1.54)	1.29 (1.21–1.38)	1.22 (1.17–1.27)
Declining eGFR slope	1.44 (1.19–1.75)	1.33 (1.22–1.45)	1.20 (1.13–1.27)	1.21 (1.17–1.25)

### Adjusted associations of positive eGFR slope

In fully adjusted multinomial logistic regression models, younger age, male gender, black race, diabetes mellitus, hypertension, cardiovascular disease, chronic lung disease, peripheral artery disease, dementia, hepatitis C, HIV, were associated with improved eGFR slope(Table 3). In models that additionally adjust for weight, weight loss was associated with higher odds of improved eGFR slope (Table 3). Inclusion of albuminuria as a covariate revealed an inverse graded association between severity of albuminuria and likelihood of improved eGFR; all other predictors exhibited results consistent with those obtained in models not adjusted for albuminuria.

**Table 3:** Adjusted associations of improved eGFR slope, and declining eGFR slopes. Model adjusted for age, race, gender, diabetes mellitus, hypertension, cardiovascular disease, hyperlipidemia, peripheral artery disease, cerebrovascular disease, chronic lung disease, hepatitis C, HIV, dementia, and initial eGFR. Reference group is patients with stable eGFR slope.

	Improved eGFR slope HR (CI)	Declining eGFR slope HR (CI)
Age	0.97 (0.97–0.97)	1.01 (1.01–1.01)
Female gender	0.82 (0.77–0.87)	1.03 (0.97–1.10)
Black race	1.12 (1.08–1.17)	1.23 (1.19–1.28)
Other race	1.01 (0.93–1.10)	1.14 (1.05–1.24)
Cerebrovascular accident	1.08 (0.96–1.22)	1.37 (1.24–1.52)
Cardiovascular disease	1.10 (1.08–1.13)	1.46 (1.42–1.49)
Dementia	1.41 (1.34–1.49)	1.29 (1.23–1.35)
Diabetes mellitus	1.15 (1.13–1.18)	1.96 (1.92–2.01)
Hepatitis C	1.40 (1.28–1.54)	1.81 (1.66–1.98)
HIV	1.25 (1.19–1.31)	1.27 (1.22–1.33)
Hypertension	1.10 (1.07–1.14)	1.88 (1.82–1.95)
Hyperlipidemia	0.97 (0.94–0.99)	1.00 (0.97–1.02)
Chronic lung disease	1.33 (1.29–1.36)	1.31 (1.28–1.35)
Peripheral artery disease	1.18 (1.12–1.24)	1.68 (1.60–1.75)
Initial eGFR	0.95 (0.95–0.96)	1.02 (1.01–1.02)
Annual percentage weight change*	0.96 (0.96–0.97)	1.00 (1.00–1.01)
Microalbuminuria**	0.94 (0.90–0.98)	1.34 (1.28–1.39)
Albuminuria**	0.76 (0.69–0.84)	2.20 (2.05–2.37)

*Model additionally adjusted for weight n = 185,508

** Model additionally adjusted for albuminuria n = 63,021

### Renal function trajectories of patients with positive eGFR slopes

**Fig 2:**
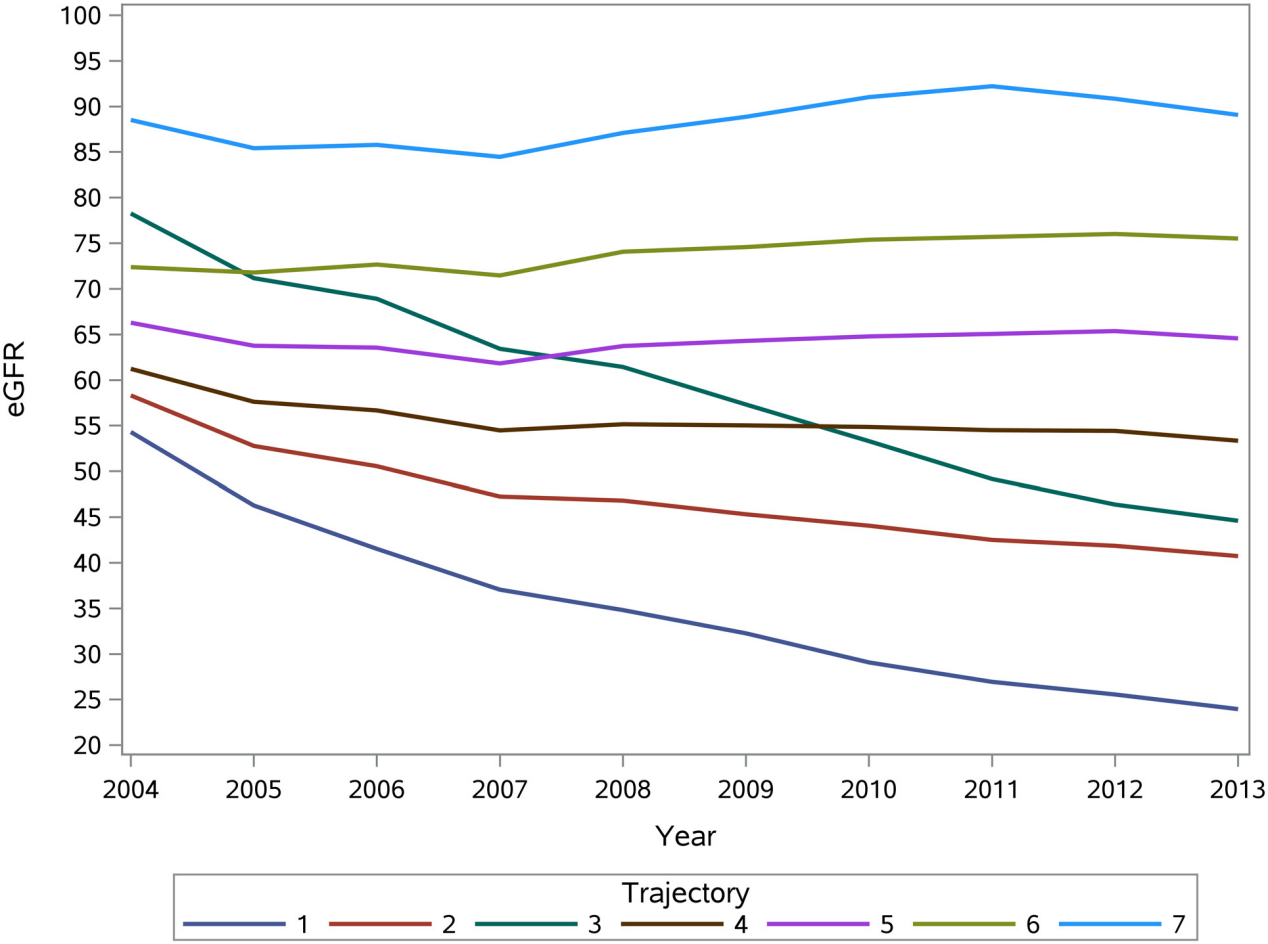
Renal function trajectories.

As described in the method section, we used Pattern-Mixture group based Latent Trajectory Models to characterize the trajectory groups of patients with improved eGFR slopes; this analysis yielded 7 trajectory groups (Fig 2).

The demographic, clinical and laboratory characteristics of patients according to renal function trajectory are presented in Table 4. Trajectory A (2.30%) and B (10.13%) were characherized by elevated eGFR (high intercept) of 88.52 and 72.39 ml/min/1.73m^2^; respectively, and annual percentage eGFR change of 1.25 and 1.56%; respectively. Trajectory C (26.88%), and D (33.16%) were charachterized by intermediate intercept of 66.29, and 61.23 ml/min/1.73m^2^; respectively, and exhibited mild downward trajectory with annual percent reduction of eGFR of -0.15%, and -2.36%; respectively. Trajectory E (18.38%), and G (4.95%) were charachterized by lower eGFR intercept of 58.3, and 54.31% ml/min/1.73m^2^; respectively, and exhibited steep downward trajectory with annual percent reduction of eGFR of -5.45%, -13.10%; respectively. Trajectory F (4.20%) was characterized by high eGFR intercept of 78.27 ml/min/1.73m^2^ and annual percent change in eGFR of -7.59%. Adjusted associations for each trajectory group are presented in S2 Table.

**Table 4:** Demographic and clinical characteristics according to renal function trajectory.

	Over all	A	B	C	D	E	F	G
Number (%)	41,410	951 (2.30)	4,194 (10.13)	11,132 (26.88)	13,731 (33.16)	7,611 (18.38)	1,741 (4.20)	2,050 (4.95)
9 year Mortality (%)	17,519 (42.31)	576 (60.57)	1,758 (41.92)	4,038 (36.27)	5,384 (39.21)	3,500 (45.99)	844 (48.48)	1,419 (69.22)
5 year Mortality (%)	10,202 (24.64)	475 (49.95)	1,012 (24.13)	2,345 (21.07)	3,073 (22.38)	1,931 (25.37)	491 (28.20)	875 (42.68)
Age (SD)	69.48 (8.53)	63.03 (10.48)	66.99 (9.40)	68.70 (8.83)	70.25 (8.12)	71.36 (7.11)	68.25 (9.06)	70.77 (7.81)
Race								
White (%)	35,481 (85.68)	674 (70.87)	3,531 (84.19)	9,656 (86.74)	11,939 (86.95)	6,603 (86.76)	1,401 (80.47)	1,677 (81.80)
Black (%)	5,205 (12.57)	265 (27.87)	578 (13.78)	1,261 (11.33)	1,562 (11.38)	893 (11.73)	310 (17.81)	336 (16.39)
Other (%)	724 (1.75)	12 (1.26)	85 (2.03)	215 (1.93)	230 (1.68)	115 (1.51)	30 (1.72)	37 (1.80)
Male Gender (%)	39,293 (94.89)	799 (84.02)	3,927 (93.63)	10,564 (94.90)	13,055 (95.08)	7,343 (96.48)	1,615 (92.76)	1,990 (97.07)
Cerebrovascular Disease (%)	400 (0.97)	13 (1.37)	34 (0.81)	75 (0.67)	131 (0.95)	84 (1.10)	30 (1.72)	33 (1.61)
Cardiovascular Disease (%)	19,772 (47.75)	429 (45.11)	1,832 (43.68)	4,909 (44.10)	6,367 (46.37)	4,103 (53.91)	874 (50.20)	1,258 (61.37)
Dementia (%)	2,212 (5.34)	83 (8.73)	235 (5.60)	556 (4.99)	700 (5.10)	367 (4.82)	122 (7.01)	149 (7.27)
Diabetes Mellitus (%)	14,639 (35.35)	376 (39.54)	1,438 (34.29)	3,412 (30.65)	4,343 (31.63)	3,178 (41.76)	812 (46.64)	1,080 (52.68)
Hepatitis C (%)	816 (1.97)	58 (6.10)	121 (2.89)	204 (1.83)	206 (1.50)	116 (1.52)	58 (3.33)	53 (2.59)
HIV (%)	2,991 (7.22)	128 (13.46)	376 (8.97)	789 (7.09)	863 (6.29)	490 (6.44)	185 (10.63)	160 (7.80)
Hypertension (%)	35,073 (84.70)	769 (80.86)	3,451 (82.28)	9,094 (81.69)	11,501 (83.76)	6,857 (90.09)	1,511 (86.79)	1,890 (92.20)
Hyperlipidemia (%)	29,554 (71.37)	602 (63.30)	2,847 (67.88)	7,939 (71.32)	9,828 (71.58)	5,588 (73.42)	1,214 (69.73)	1,536 (74.93)
Chronic Lung Disease (%)	11,283 (27.25)	378 (39.75)	1,285 (30.64)	2,904 (26.09)	3,463 (25.22)	2,084 (27.38)	545 (31.30)	624 (30.44)
Peripheral Artery Disease (%)	2,166 (5.23)	86 (9.04)	210 (5.01)	466 (4.19)	617 (4.49)	168 (6.15)	129 (7.41)	190 (9.27)
Average eGFR slope before T0 ml/min/1.73m2/year (SD)	4.01 (3.36)	8.19 (7.88)	5.01 (3.76)	4.06 (2.96)	3.59 (2.64)	3.39 (2.84)	6.01 (4.93)	3.17 (2.86)
Average initial eGFR in ml/min/1.73m2 (SD)	53.18 (4.30)	54.88 (3.96)	55.00 (3.88)	54.41 (3.96)	52.59 (4.17)	51.44 (4.21)	55.05 (3.85)	50.83 (4.17)
Average T0 eGFR in ml/min/1.73m2 (SD)	64.18 (10.29)	88.52 (13.73)	72.39 (9.24)	66.29 (7.08)	61.23 (6.49)	58.31 (7.59)	78.27 (11.52)	54.31 (10.44)
Median duration between initial and T0 eGFR in years (IQR)	3.51 (2.43, 4.26)	4.01 (3.16, 4.50)	3.72 (2.74, 4.36)	3.51 (2.49, 4.23)	3.36 (2.28, 4.20)	3.44 (2.32, 4.24)	3.71 (2.71, 4.36)	3.71 (2.53, 4.38)
Median number of eGFR measure before T0 (IQR)	8 (5, 12)	12 (7, 18)	9 (6, 13)	8 (5, 11)	7 (5, 11)	8 (5, 13)	9 (6, 14)	10 (6, 16)
Median number of eGFR measure after T0 (IQR)	13 (7, 21)	12 (5, 22)	14 (7, 22)	13 (7, 21)	13 (7, 21)	14 (7, 22)	16 (9, 26)	12 (6, 24)
Average eGFR change after T0 in ml/min/1.73m^2^/year (SD)	-1.82 (7.20)	0.31 (12.18)	0.82 (6.66)	-0.36 (6.55)	-1.66 (5.85)	-3.39 (6.60)	-6.19 (8.73)	-7.53 (10.92)
Average Annual percentage eGFR change after T0 (SD)	-2.63% (11.36%)	1.25% (14.49%)	1.56% (9.54%)	-0.15% (9.95%)	-2.36% (9.41%)	-5.45% (11.21%)	-7.59% (9.97%)	-13.10% (20.02%)
Weight								
Number	38997	779	4,071	10,719	12,941	7,007	1,592	1,888
Average Annual change lb/year (SD)	-1.20 (7.01)	-2.99 (8.85)	-1.56 (7.43)	-1.00 (7.55)	-1.03 (5.96)	-1.15 (6.35)	-2.30 (8.25)	-1.18 (9.30)
Median Annual percentage change (IQR)	-0.47% (-1.76%, 0.72%)	-1.25% (-3.20%, 0.52%)	-0.58% (-2.02%, 0.73%)	-0.41% (-1.66%, 0.75%)	-0.44% (-1.65%, 0.73%)	-0.42% (-1.70%, 0.71%)	-0.77% (-2.27%, 0.38%)	-0.54% (-2.11%, 0.78%)
Micro albumin / Creatinine Ratio								
Number	12,792	196	1,305	3,361	4,046	2,600	602	682
Normal (%)	7,716 (60.32)	124 (63.27)	882 (67.59)	2,291 (68.16)	2,521 (62.31)	1,344 (51.69)	312 (51.83)	242 (35.48)
Mild (%)	4,455 (34.83)	69 (35.20)	384 (29.43)	986 (29.34)	1,395 (34.48)	1,060 (40.77)	243 (40.37)	318 (46.63)
Severe (%)	621 (4.85)	3 (1.53)	39 (2.99)	84 (2.50)	130 (3.21)	196 (7.54)	47 (7.81)	122 (17.89)

We then examined the risk of death in the different renal function trajectories at 1, 3, 5, and 9 years. Compared to the referrant group (patients with stable eGFR slope before T0), the risk of death at 9 years was significantly elevated in trajectory A (HR = 4.49; CI: 4.10–4.91), and B (HR = 1.46; CI: 1.39–1.54). Patients in trajectories C, D, E exhibited mildly increased risk of death, while patients in trajectories F and G had siginificantly increased risk of death at 9 yeas (HR = 1.61; CI:1.49–1.73, and HR = 2.10; CI:1.99–2.22; respectively) (Table 5). Addition of weight and albuminuria in the models yeilded consistent results (S3a and S3b Table). The association between trajectory E, and G and the risk of death at 9 years was attenuated due to presence and severity of albuminuria; p for interaction was 0.0343, and 0.0008, respectively.

**Table 5:** Risk of death by trajectory. Model adjusted for age, race, gender, for age, race, gender, diabetes mellitus, hypertension, cardiovascular disease, hyperlipidemia, peripheral artery disease, cerebrovascular disease, chronic lung disease, hepatitis C, HIV, dementia, and eGFR at time of cohort entry (T0). Reference group is patients with stable kidney function before cohort entry (T0).

	1-year HR (CI)	3-year HR (CI)	5-year HR (CI)	9-year HR (CI)
A	8.66 (7.34–10.23)	6.08 (5.38–6.87)	5.45 (4.91–6.05)	4.49 (4.10–4.91)
B	1.37 (1.18–1.60)	1.41 (1.29–1.54)	1.47 (1.37–1.58)	1.46 (1.39–1.54)
C	1.45 (1.32–1.60)	1.35 (1.27–1.43)	1.25 (1.19–1.31)	1.14 (1.10–1.19)
D	1.55 (1.43–1.68)	1.30 (1.23–1.36)	1.21 (1.16–1.26)	1.12 (1.09–1.15)
E	1.73 (1.58–1.90)	1.28 (1.21–1.37)	1.23 (1.17–1.29)	1.20 (1.16–1.24)
F	2.66 (2.25–3.14)	1.78 (1.58–2.01)	1.65 (1.50–1.81)	1.61 (1.49–1.73)
G	2.54 (2.22–2.90)	2.04 (1.87–2.22)	2.06 (1.93–2.21)	2.10 (1.99–2.22)

**Fig 3:**
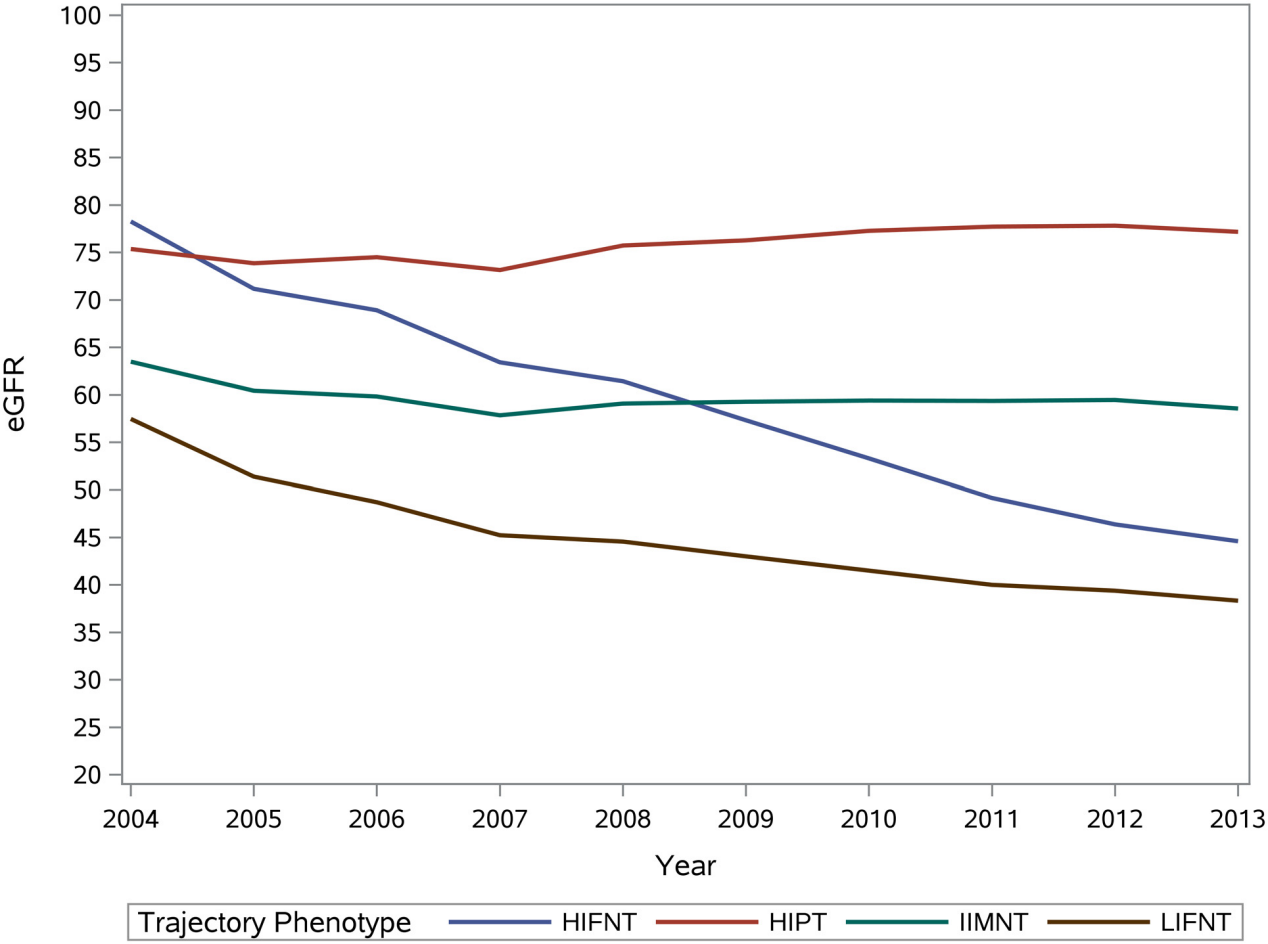
Renal function trajectory phenotypes.

Further phenotypic characherization of trajectory groups based on intercept (high, intermediate, low) and average annual percent eGFR change (positive, mild negative, fast negative eGFR change) yielded 4 distinct categories: High Intercept Positive Trajectory (HIPT) includes trajectory A and B; Intermediate Intercept Mild Negative Trajectory (IIMNT) includes trajectory C, and D; Low Intercept Fast Negative Trajectory (LIFNT) includes trajectory E and G; and High Intercept and Fast Negative Trajectory (HIFNT) which includes trajectory F (Fig 3).

Adjusted associations of these phenotypes are presented in Table 6 and show that compared to IIMNT (reference group), HIPT is associated with younger age, dementia, HIV, chronic lung disease, peripheral artery disease, weight loss, and inversely associated with albuminuria while diabetes, hypertension, carrdiovascular disease, peripheral artery disease, and albuminuria are asscoiated with LIFNT and HIFNT (Table 6). The risk of death at 1, 3, 5, 9 years was lowest in IIMNT, and highest in HIPT (Table 7 and S4a and S4b Table). The presence and severity of albuminuria attentuated the asscoation between trajectory phenotype LIFNT and risk of death (p for interaction = 0.0215). Formal interaction analyses were non significant for other trajectory phenotypes.

**Table 6:** Adjusted associations of trajectory phenotypes. HIFNT = High Intercept and Fast Negative Trajectory includes trajectory F. HIPT = High Intercept Positive Trajectory and includes trajectory A and B; IIMNT = Intermediate Intercept Mild Negative Trajectory and includes trajectory C, and D; LIFNT = Low Intercept Fast Negative Trajectory and includes trajectory E and G; Model adjusted for age, race, gender, for age, race, gender, diabetes mellitus, hypertension, cardiovascular disease, hyperlipidemia, peripheral artery disease, cerebrovascular disease, chronic lung disease, hepatitis C, HIV, dementia, and eGFR at time of cohort entry (T0). Reference group is IIMNT.

	HIFNT OR (CI)	HIPT OR (CI)	IIMNT OR	LIFNT OR (CI)
Age	1.00 (0.99–1.00)	0.97 (0.96–0.97)	1	1.03 (1.02–1.03)
Female gender	0.90 (0.70–1.17)	0.92 (0.79–1.08)	1	0.89 (0.77–1.03)
Black race	1.13 (0.96–1.32)	1.00 (0.90–1.11)	1	1.26 (1.16–1.37)
Other race	0.94 (0.63–1.41)	0.96 (0.74–1.23)	1	0.95 (0.78–1.17)
Cerebrovascular accident	1.69 (1.08–2.62)	1.01 (0.70–1.46)	1	1.08 (0.84–1.38)
Cardiovascular disease	1.10 (0.99–1.23)	0.98 (0.91–1.06)	1	1.22 (1.16–1.29)
Dementia	1.18 (0.95–1.46)	1.21 (1.04–1.40)	1	0.87 (0.77–0.98)
Diabetes mellitus	1.58 (1.42–1.76)	1.07 (0.99–1.15)	1	1.59 (1.51–1.67)
Hepatitis C	1.26 (0.91–1.74)	1.16 (0.93–1.45)	1	1.21 (0.99–1.48)
HIV	1.37 (1.14–1.65)	1.20 (1.06–1.36)	1	0.92 (0.83–1.02)
Hypertension	1.22 (1.04–1.43)	1.02 (0.93–1.12)	1	1.50 (1.38–1.63)
Hyperlipidemia	0.95 (0.84–1.08)	0.91 (0.84–0.98)	1	0.95 (0.89–1.00)
Chronic lung disease	1.01 (0.95–1.21)	1.26 (1.16–1.36)	1	1.01 (0.95–1.07)
Peripheral artery disease	1.32 (1.07–1.64)	1.20 (1.02–1.40)	1	1.23 (1.10–1.37)
T0 eGFR	1.19 (1.18–1.19)	1.16 (1.15–1.16)	1	0.90 (0.90–0.90)
Annual percentage weight change *	0.98 (0.97–1.00)	0.99 (0.98–1.00)	1	1.00 (0.99–1.00)
Microalbuminuria**	1.41 (1.17–1.71)	0.89 (0.78–1.03)	1	1.57 (1.43–1.73)
Albuminuria**	2.65 (1.82–3.86)	0.76 (0.52–1.09)	1	4.33 (3.55–5.27)

*Model additionally adjusted for weight n = 38,997

** Model additionally adjusted for albuminuria n = 12,736

**Table 7:** Risk of death of trajectory phenotypes. HIFNT = High Intercept and Fast Negative Trajectory includes trajectory F. HIPT = High Intercept Positive Trajectory and includes trajectory A and B; IIMNT = Intermediate Intercept Mild Negative Trajectory and includes trajectory C, and D; LIFNT = Low Intercept Fast Negative Trajectory and includes trajectory E and G; Model adjusted for age, race, gender, for age, race, gender, diabetes mellitus, hypertension, cardiovascular disease, hyperlipidemia, peripheral artery disease, cerebrovascular disease, chronic lung disease, hepatitis C, HIV, dementia, and eGFR at time of cohort entry (T0). Reference group is patients with stable kidney function before T0.

	1-year HR (CI)	3-year HR (CI)	5-year HR (CI)	9-year HR (CI)
HIFNT	2.25 (1.91–2.65)	1.64 (1.45–1.84)	1.56 (1.42–1.72)	1.56 (1.45–1.68)
HIPT	2.23 (1.98–2.50)	1.88 (1.74–2.03)	1.83 (1.72–1.95)	1.71 (1.63–1.79)
IIMNT	1.41 (1.32–1.51)	1.28 (1.22–1.33)	1.20 (1.16–1.24)	1.12 (1.09–1.14)
LIFNT	1.86 (1.72–2.01)	1.43 (1.36–1.50)	1.39 (1.34–1.45)	1.36 (1.32–1.40)

### Sensitivity analyses

To ascertain the validity of the results, we undertook a number of sensitivity analyses (as described in the Methods section) and results were consistent with those shown in the primary analyses.

## Discussion

Our results suggests that patients with early CKD and positive eGFR slope (or improved kidney function) follow a number of distinct trajectory types when observed over a long period of time. Some of them follow a trajectory characterized by high eGFR intercept and positive trajectory over the ensuing years (HIPT = trajectory A, B), some follow a trajectory with relatively high eGFR intercept followed by fast negative trajectory (HIFNT = trajectory F), and some have lower eGFR at intercept and exhibit fast negative trajectory over time (LIFNT = trajectory E, G). Examination of adjusted associactions reveals that dementia, chronic lung disease, and changes in weight were strong predictors of trajectory phenotypes characterized by elevated intercept and mild positive eGFR trend (HIPT), while traditional risk factors—generally associated with CKD prevalance and progression-including diabetes mellitus, hypertension, albuminuria, and cardiovascular disease were strong predictors of trajectory phenotypes characterized by significant annual percent decline in eGFR (LIFNT, HIFNT).

The increased risk of death in patients with positive eGFR slope and subsequent HIPT phenotype is not fully explained by changes in weight; addition of weight to the models did not change the risk estimate significantly. Since our study did not account for changes in body composition, we cannot completely rule out changes in eGFR that might be due to loss of muscle mass. However, conditions associated with fraility including dementia, and chronic lung disease showed significnat association with specific trajectories (A, B), and it is likely that increased eGFR in these patients is the result of decreased muscle mass generally associated with frailty or residual confouding related to the condition of dementia, or chronic lung disesase which are not captured in our analyses.

Consistent evidence from different groups suggests that high eGFR or hyperfiltration is associated with increased risk of death, and that this relationship is more pronounced in patients with proteinuria [22–24]. In our studies, HIPT (trajectory A and B) was associated with increased risk of death, but showed no significant association with correlates of hyperfiltration (diabetes mellitus, and albuminuria), and albuminuria did not modify the risk of death (insignificant interaction). Taken together, the observations suggest that hyperfiltration—while hypothetically plausible-is not a likely explanation for the increased risk of death of patients in this phenotype.

The presence and severity of albuminuria was strongly associated with trajectory phenotypes that are characterized by fast negative eGFR change over time, and high risk of death. Formal interaction analyses show that presence and severity of albuminuria attenuated the risk of death, most likely a reflection of the intimate relationship of albuminuria with faster decline. Conversely, in the absence of albuminuria, the relationship is strong and significant, suggesting that eGFR changes captured by slope analyses, or trajectory phenotyping, contribute meanigfully to risk in patients with early CKD.

Our study has several limitations. The cohort included mostly white males, thus the results may not be generalizable to less narrowly defined populations. The imperfect nature of administrative data and the retrospective design of the study may also lead to sampling bias and inaccurate measurements of the predictor variables. In order to minimize such measurement bias, we used published definitions of comorbid illnesses that are validated for use in administrative data. Because of inclusion criteria specifying the minimum number of creatinine measurements required and since the frequency of creatinine measurements is probably a surrogate marker of poorer overall health, we may have systematically missed those who rarely seek care within the VA and our cohort may be sicker than a broader population of veterans.

That positive eGFR slope is associated with increased risk of death is counterintuitive albeit a consistent finding in several independent reports [2, 3, 5, 6]. To our knowledge, however, this is the first study to examine characteristics of patients with positive eGFR slopes and follow them longitudinally to describe their long term renal function trajectories and characterize the relationship between trajectory type and risk of death. While our suggests some answers, more studies are needed to further evaluate long term renal function in those with positive eGFR slopes and develop a better understanding of the mechanism(s) underpinning the association between longitudinal kidney function parameters and risk of death.

Evaluation of longitudinal eGFR is very valuable in informing risk estimation of untoward events in patients with CKD [25–28]. However, important challenges remain in that there is significant heterogeneity in methods used to estimate slopes (or eGFR change over time); and none are without limitations. Comparative evaluation and validation of different methods to capture eGFR changes over time is needed. Trajectory modeling, an approach that incorporates all available data points to construct an individual patient renal trajectory, maybe a more nuanced method to capture longitudinal eGFR changes over time and inform risk estimation of untoward events. Further studies are needed to validate this approach in different cohorts, and across the spectrum of CKD. The increased availability of electronic medical record systems, and the nearly ubiquitous presence of computing devices (including mobile technologies) in the clinical environment present an opportunity for rapid application and scaling of such an approach-when validated-to inform decision support systems at the population and patient levels, allocation of finite healthcare resources, and prognostication at individual patient level.

## Supporting Information

S1 Fig Distribution of eGFR slopes. (TIFF)

S1 Table Change in kidney function and the risk of death controlling for initial eGFR. (DOCX)

S2 Table Adjusted associations of trajectory types. (DOCX)

S3 Table Risk of death by trajectory. (DOCX)

S4 Table Risk of death of trajectory phenotypes. (DOCX)
