# Supplementary figures and images for: Renal Function Trajectories in Patients with Prior Improved eGFR Slopes and Risk of Death
Source: PLoS One. 2016 Feb 22;11(2):e0149283. doi: 10.1371/journal.pone.0149283 (PMC4762675; doi:10.1371/journal.pone.0149283)

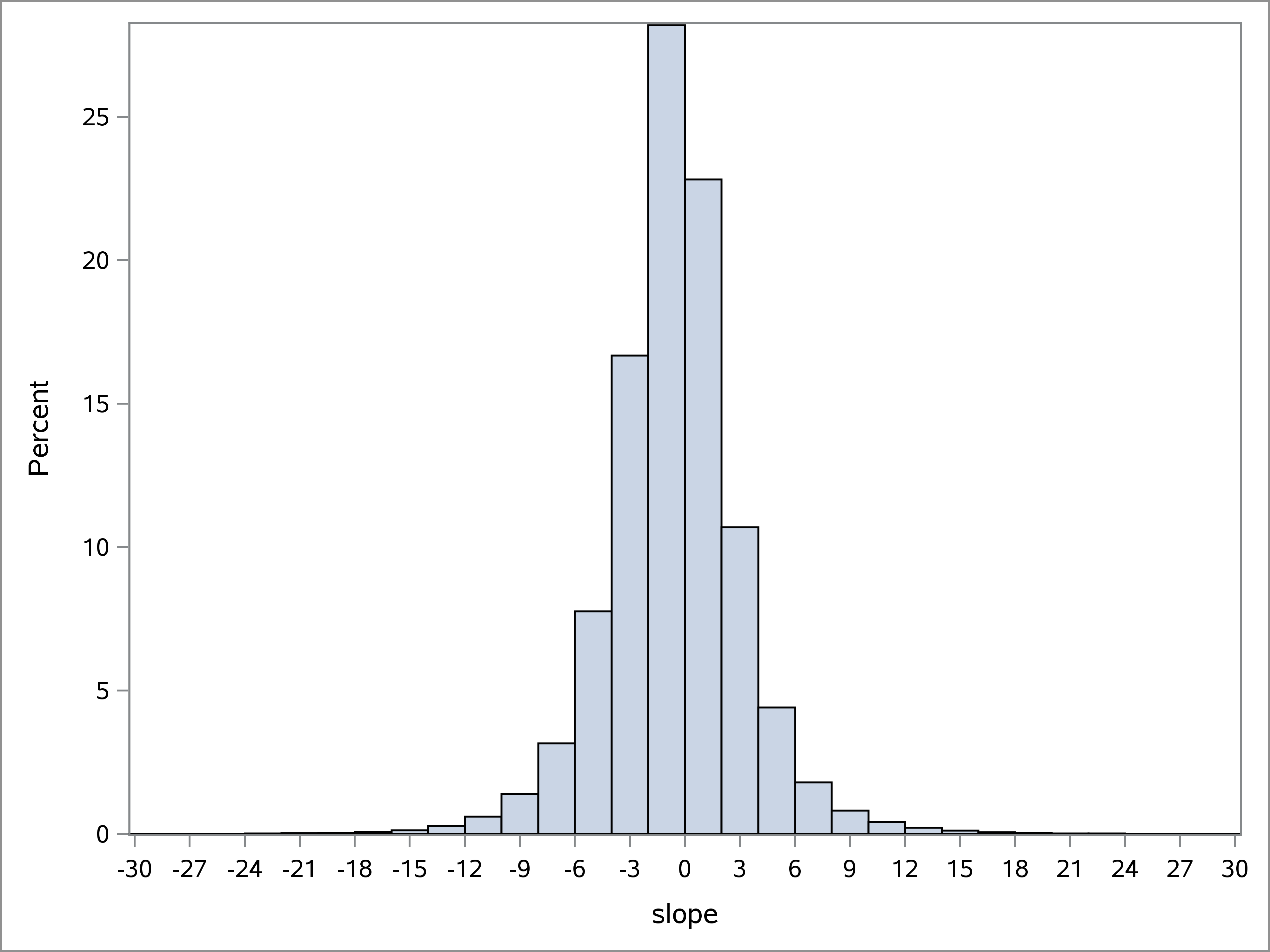

Supplement: S1 Fig — (TIFF) [file pone.0149283.s001.tiff]
