# Supplementary material for: Renal Function Trajectories in Patients with Prior Improved eGFR Slopes and Risk of Death
Source: PLoS One. 2016 Feb 22;11(2):e0149283. doi: 10.1371/journal.pone.0149283 (PMC4762675; doi:10.1371/journal.pone.0149283)
Supplement: S1 Table — (DOCX) [file pone.0149283.s002.docx]

**S1 Table:**  **Change in kidney function and the risk of death controlling for initial eGFR**

|  | 1-year HR  (CI) | 3-year HR  (CI) | 5-year HR  (CI) | 9-year HR  (CI) |
| --- | --- | --- | --- | --- |
| Model 1 | | | | |
| Improved eGFR slope | 1.79  (1.71-1.88) | 1.38  (1.34-1.42) | 1.27  (1.24-1.30) | 1.17  (1.15-1.19) |
| Declining eGFR slope | 2.47  (2.37-2.58) | 1.84  (1.79-1.88) | 1.66  (1.63-1.69) | 1.54  (1.51-1.56) |
| Model 1 + annual percentage weight change | | | | |
| Improved eGFR slope | 1.50  (1.40-1.60) | 1.26  (1.22-1.30) | 1.19  (1.16-1.22) | 1.13  (1.10-1.15) |
| Declining eGFR slope | 1.86  (1.76-1.97) | 1.61  (1.56-1.65) | 1.52  (1.49-1.56) | 1.46  (1.43-1.48) |
| Model 1 + annual percentage weight change + albuminuria | | | | |
| Improved eGFR slope | 1.34  (1.09-1.65) | 1.15  (1.05-1.26) | 1.09  (1.02-1.15) | 1.07  (1.03-1.11) |
| Declining eGFR slope | 1.93  (1.63-2.27) | 1.65  (1.53-1.77) | 1.45  (1.38-1.52) | 1.39  (1.35-1.43) |
| Model 1 adjusted for age, race, gender, diabetes mellitus, hypertension, cardiovascular disease, hyperlipidemia, peripheral artery disease, cerebrovascular disease, chronic lung disease, hepatitis C, HIV, dementia and initial eGFR.  Reference group is patients with stable kidney function. | | | | |
