# Supplementary material for: Renal Function Trajectories in Patients with Prior Improved eGFR Slopes and Risk of Death
Source: PLoS One. 2016 Feb 22;11(2):e0149283. doi: 10.1371/journal.pone.0149283 (PMC4762675; doi:10.1371/journal.pone.0149283)
Supplement: S2 Table — (DOCX) [file pone.0149283.s003.docx]

**S2 Table: Adjusted associations of trajectory types.**

|  | Trajectory A  OR  (CI) | Trajectory B  OR  (CI) | Trajectory C  OR  (CI) | Trajectory D  OR | Trajectory E  OR  (CI) | Trajectory F  OR  (CI) | Trajectory G  OR  (CI) |
| --- | --- | --- | --- | --- | --- | --- | --- |
| Age | 0.93  (0.92-0.94) | 0.96  (0.95-0.96) | 0.98  (0.97-0.98) | 1 | 1.02  (1.01-1.02) | 0.98  (0.97-0.98) | 1.01  (1.00-1.02) |
| Female gender | 0.49  (0.35-0.70) | 0.83  (0.69-0.99) | 0.82  (0.72-0.94) | 1 | 0.91  (0.77-1.06) | 0.97  (0.74-1.27) | 0.56  (0.41-0.75) |
| Black race | 0.96  (0.76-1.21) | 0.89  (0.79-1.01) | 0.87  (0.80-0.95) | 1 | 1.13  (1.03-1.24) | 1.13  (0.95-1.33) | 1.56  (1.35-1.81) |
| Other race | 0.71  (0.37-1.37) | 1.02  (0.77-1.36) | 1.05  (0.86-1.27) | 1 | 0.93  (0.74-1.17) | 0.93  (0.61-1.42) | 1.15  (0.80-1.66) |
| Cerebrovascular accident | 1.15  (0.57-2.31) | 0.82  (0.54-1.26) | 0.75  (0.55-1.01) | 1 | 0.94  (0.71-1.25) | 1.46  (0.90-2.37) | 1.14  (0.76-1.72) |
| Cardiovascular disease | 1.05  (0.89-1.25) | 0.96  (0.88-1.04) | 0.97  (0.92-1.02) | 1 | 1.17  (1.10-1.25) | 1.09  (0.97-1.23) | 1.39  (1.26-1.55) |
| Dementia | 1.99  (1.48-2.68) | 1.13  (0.96-1.34) | 1.03  (0.91-1.16) | 1 | 0.83  (0.73-0.95) | 1.25  (0.99-1.57) | 1.11  (0.91-1.36) |
| Diabetes mellitus | 1.15  (0.97-1.36) | 1.01  (0.93-1.10) | 0.93  (0.88-0.98) | 1 | 1.46  (1.37-1.55) | 1.50  (1.34-1.69) | 2.02  (1.83-2.23) |
| Hepatitis C | 1.30  (0.86-1.98) | 1.13  (0.87-1.48) | 1.00  (0.81-1.23) | 1 | 1.12  (0.88-1.42) | 1.30  (0.91-1.85) | 1.58  (1.13-2.21) |
| HIV | 1.45  (1.12-1.87) | 1.27  (1.10-1.46) | 1.12  (1.00-1.24) | 1 | 0.96  (0.85-1.08) | 1.49  (1.23-1.81) | 0.99  (0.82-1.20) |
| Hypertension | 0.93  (0.75-1.17) | 0.99  (0.89-1.10) | 0.93  (0.87-1.00) | 1 | 1.47  (1.35-1.61) | 1.14  (0.97-1.35) | 1.46  (1.23-1.74) |
| Hyperlipedemia | 0.95  (0.79-1.14) | 0.92  (0.85-1.01) | 1.04  (0.97-1.10) | 1 | 0.96  (0.90-1.02) | 0.98  (0.86-1.11) | 0.96  (0.85-1.07) |
| Chronic lung disease | 1.75  (1.48-2.08) | 1.26  (1.16-1.38) | 1.07  (1.01-1.14) | 1 | 1.03  (0.97-1.10) | 1.14  (1.00-1.29) | 1.06  (0.95-1.18) |
| Peripheral artery disease | 1.85  (1.38-2.49) | 1.08  (0.90-1.30) | 0.97  (0.85-1.11) | 1 | 1.16  (1.02-1.32) | 1.36  (1.08-1.72) | 1.42  (1.18-1.71) |
| T0 eGFR | 1.36  (1.35-1.38) | 1.21  (1.20-1.22) | 1.11  (1.10-1.11) | 1 | 0.94  (0.94-0.95) | 1.28  (1.27-1.29) | 0.88  (0.88-0.89) |
|  | | | | | | | |
| Weight change* | 0.96  (0.94-0.98) | 0.99  (0.98-1.00) | 1.00  (1.00-1.01) | 1 | 1.00  (0.99-1.01) | 0.98  (0.96-1.00) | 1.00  (0.99-1.02) |
|  | | | | | | | |
| Microalbuminuria** | 0.87  (0.61-1.24) | 0.78  (0.67-0.90) | 0.80  (0.72-0.89) | 1 | 1.34  (1.20-1.49) | 1.24  (1.02-1.52) | 2.12  (1.76-2.55) |
| Albuminuria** | 0.28  (0.08-0.94) | 0.62  (0.41-0.92) | 0.64  (0.47-0.85) | 1 | 2.79  (2.20-3.53) | 1.94  (1.29-2.91) | 9.23  (6.86-12.41) |
|  | | | | | | | |
| Model adjusted for age, race, gender, for age, race, gender, diabetes mellitus, hypertension, cardiovascular disease, hyperlipidemia, peripheral artery disease, cerebrovascular disease, chronic lung disease, hepatitis C, HIV, dementia, and eGFR at time of cohort entry (time zero).  Reference group is patients with mild eGFR decline after having eGFR improvement.  *model additionally adjusted for weight n=38,997  ** model additionally adjusted for albuminuria n=12,736 | | | | | | | |
