# Supplementary material for: Renal Function Trajectories in Patients with Prior Improved eGFR Slopes and Risk of Death
Source: PLoS One. 2016 Feb 22;11(2):e0149283. doi: 10.1371/journal.pone.0149283 (PMC4762675; doi:10.1371/journal.pone.0149283)
Supplement: S3 Table — (DOCX) [file pone.0149283.s004.docx]

**S3 table a: Risk of death by trajectory, additionally control for annual percentage weight change:**

|  | 1-year HR  (CI) | 3-year HR  (CI) | 5-year HR  (CI) | 9-year HR  (CI) |
| --- | --- | --- | --- | --- |
|  | | | | |
| A | 7.65  (6.02-9.72) | 4.76  (4.09-5.53) | 4.34  (3.83-4.92) | 3.61  (3.25-4.01) |
| B | 1.58  (1.31-1.91) | 1.47  (1.33-1.62) | 1.51  (1.40-1.63) | 1.48  (1.40-1.56) |
| C | 1.44  (1.27-1.64) | 1.33  (1.25-1.42) | 1.23  (1.17-1.29) | 1.13  (1.09-1.17) |
| D | 1.53  (1.38-1.70) | 1.26  (1.19-1.33) | 1.17  (1.12-1.22) | 1.09  (1.06-1.13) |
| E | 1.07  (0.93-1.25) | 1.07  (0.99-1.15) | 1.11  (1.06-1.17) | 1.14  (1.10-1.18) |
| F | 1.51  (1.14-2.00) | 1.28  (1.10-1.49) | 1.34  (1.20-1.50) | 1.43  (1.32-1.55) |
| G | 2.33  (1.95-2.79) | 1.94  (1.76-2.14) | 2.01  (1.87-2.16) | 2.07  (1.95-2.18) |
| Model adjusted for age, race, gender, for age, race, gender, diabetes mellitus, hypertension, cardiovascular disease, hyperlipidemia, peripheral artery disease, cerebrovascular disease, chronic lung disease, hepatitis C, HIV, dementia, eGFR at time of cohort entry (time zero) and annual percentage weight change.  Reference group is patients with stable kidney function before cohort entry (time zero). | | | | |

**S3 table b: Risk of death by trajectory, additionally control for annual percentage weight change and albuminuria**

|  | 1-year HR  (CI) | 3-year HR  (CI) | 5-year HR  (CI) | 9-year HR  (CI) |
| --- | --- | --- | --- | --- |
|  | | | | |
| A | 13.48  (6.21-29.23) | 3.98  (2.44-6.51) | 3.82  (2.71-5.40) | 2.66  (2.06-3.44) |
| B | 1.87  (1.01-3.45) | 1.36  (1.02-1.80) | 1.37  (1.14-1.65) | 1.32  (1.17-1.48) |
| C | 1.68  (1.12-2.51) | 1.18  (0.98-1.43) | 1.11  (0.98-1.26) | 1.02  (0.94-1.10) |
| D | 1.58  (1.13-2.21) | 1.28  (1.10-1.48) | 1.09  (0.98-1.21) | 1.05  (0.99-1.12) |
| E | 1.04  (0.67-1.60) | 1.07  (0.90-1.28) | 1.06  (0.95-1.20) | 1.10  (1.03-1.18) |
| F | 1.50  (0.63-3.54) | 0.90  (0.58-1.38) | 1.06  (0.81-1.38) | 1.30  (1.12-1.51) |
| G | 1.84  (1.05-3.23) | 1.90  (1.50-2.40) | 2.08  (1.79-2.42) | 1.93  (1.74-2.14) |
| Model adjusted for age, race, gender, for age, race, gender, diabetes mellitus, hypertension, cardiovascular disease, hyperlipidemia, peripheral artery disease, cerebrovascular disease, chronic lung disease, hepatitis C, HIV, dementia, eGFR at time of cohort entry (time zero), annual percentage weight change and albuminuria  Reference group is patients with stable kidney function before cohort entry (time zero). | | | | |
