# Supplementary material for: Renal Function Trajectories in Patients with Prior Improved eGFR Slopes and Risk of Death
Source: PLoS One. 2016 Feb 22;11(2):e0149283. doi: 10.1371/journal.pone.0149283 (PMC4762675; doi:10.1371/journal.pone.0149283)
Supplement: S4 Table — (DOCX) [file pone.0149283.s005.docx]

**S4 table a: Risk of death of trajectory phenotypes, additionally control for annual percentage weight change**

|  | 1-year HR  (CI) | 3-year HR  (CI) | 5-year HR  (CI) | 9-year HR  (CI) |
| --- | --- | --- | --- | --- |
|  | | | | |
| HIFNT | 1.36  (1.03-1.81) | 1.22  (1.05-1.42) | 1.30  (1.16-1.45) | 1.41  (1.30-1.52) |
| HIPT | 2.24  (1.91-2.63) | 1.79  (1.64-1.95) | 1.76  (1.65-1.88) | 1.65  (1.56-1.73) |
| IIMNT | 1.43  (1.31-1.57) | 1.26 (1.21-1.33) | 1.18  (1.14-1.22) | 1.10  (1.07-1.13) |
| LIFNT | 1.34  (1.19-1.51) | 1.25  (1.18-1.33) | 1.29  (1.24-1.35) | 1.31  (1.27-1.35) |
| HIFNT=High Intercept and Fast Negative Slope includes trajectory F.  HIPT=High Intercept Positive Slope and includes trajectory A and B;  IIMNT=Intermediate Intercept Mild Negative Slope and includes trajectory C, and D;  LIFNT=Low Intercept Fast Negative Slope and includes trajectory E and G;  Model adjusted for age, race, gender, for age, race, gender, diabetes mellitus, hypertension, cardiovascular disease, hyperlipidemia, peripheral artery disease, cerebrovascular disease, chronic lung disease, hepatitis C, HIV, dementia, eGFR at time of cohort entry (time zero), and annual percentage weight change.  Reference group is patients with stable kidney function before T0. | | | | |

**S 4 table b: Risk of death of trajectory phenotypes, additionally control for annual percentage weight change and albuminuria**

|  | 1-year HR  (CI) | 3-year HR  (CI) | 5-year HR  (CI) | 9-year HR  (CI) |
| --- | --- | --- | --- | --- |
|  | | | | |
| HIFNT | 1.37  (0.58-3.24) | 0.89  (0.58-1.37) | 1.05  (0.81-1.37) | 1.30  (1.12-1.51) |
| HIPT | 2.74  (1.65-4.56) | 1.61  (1.24-2.08) | 1.59  (1.34-1.88) | 1.43  (1.29-1.60) |
| IIMNT | 1.56  (1.17-2.08) | 1.24  (1.09-1.41) | 1.10  (1.01-1.20) | 1.04  (0.99-1.10) |
| LIFNT | 1.21  (0.85-1.73) | 1.26  (1.09-1.46) | 1.28 (1.17-1.41) | 1.26  (1.18-1.34) |
| HIFNT=High Intercept and Fast Negative Slope includes trajectory F.  HIPT=High Intercept Positive Slope and includes trajectory A and B;  IIMNT=Intermediate Intercept Mild Negative Slope and includes trajectory C, and D;  LIFNT=Low Intercept Fast Negative Slope and includes trajectory E and G;  Model adjusted for age, race, gender, for age, race, gender, diabetes mellitus, hypertension, cardiovascular disease, hyperlipidemia, peripheral artery disease, cerebrovascular disease, chronic lung disease, hepatitis C, HIV, dementia, eGFR at time of cohort entry (time zero), annual percentage weight change and albuminuria  Reference group is patients with stable kidney function before T0. | | | | |
